# Water-Food-Nutrition-Health Nexus: Linking Water to Improving Food, Nutrition and Health in Sub-Saharan Africa

**DOI:** 10.3390/ijerph13010107

**Published:** 2016-01-06

**Authors:** Tafadzwanashe Mabhaudhi, Tendai Chibarabada, Albert Modi

**Affiliations:** Department of Crop Science, School of Agricultural, Earth and Environmental Sciences, College of Agriculture, Engineering and Science, University of KwaZulu-Natal, Private Bag X01, Scottsville 3201, Pietermaritzburg, South Africa; tendaipolite@gmail.com (T.C.); modiat@ukzn.ac.za (A.M.)

**Keywords:** agrobiodiversity, food and nutrition security, nutritional water productivity, water-food-nutrition-health nexus

## Abstract

Whereas sub-Saharan Africa’s (SSA) water scarcity, food, nutrition and health challenges are well-documented, efforts to address them have often been disconnected. Given that the region continues to be affected by poverty and food and nutrition insecurity at national and household levels, there is a need for a paradigm shift in order to effectively deliver on the twin challenges of food and nutrition security under conditions of water scarcity. There is a need to link water use in agriculture to achieve food and nutrition security outcomes for improved human health and well-being. Currently, there are no explicit linkages between water, agriculture, nutrition and health owing to uncoordinated efforts between agricultural and nutrition scientists. There is also a need to develop and promote the use of metrics that capture aspects of water, agriculture, food and nutrition. This review identified nutritional water productivity as a suitable index for measuring the impact of a water-food-nutrition-health nexus. Socio-economic factors are also considered as they influence food choices in rural communities. An argument for the need to utilise the region’s agrobiodiversity for addressing dietary quality and diversity was established. It is concluded that a model for improving nutrition and health of poor rural communities based on the water-food-nutrition-health nexus is possible.

## 1. Introduction

“Food and nutrition security exists when all people, at all times, have physical and economic access to sufficient, safe and nutritious food that meets their dietary needs and food preferences for an active and healthy life” [1]. Over the past decades, issues of food and nutrition security have taken centre stage in defining the development agenda in sub-Saharan Africa (SSA) and other developing regions. Significant funding has been channelled towards fighting food and nutrition insecurity. Although considerable progress has been made towards combating food and nutrition insecurity on a global scale [2], the same cannot be said for SSA. At 23.8% [3], the region still has the highest prevalence of undernourishment in its population [2]. Most countries in SSA are still characterised as food and nutrition insecure [3]. Therefore, despite achievements realised over the period under review, food and nutrition security remain as a major challenge [4].

Water for agriculture and crop productivity play a significant role in delivering food and nutrition security goals [3,4]. This realisation has stimulated discussions around the water-food nexus. Within the context of SSA, it is important to note that 70% of the population relies on agriculture [5], either directly or indirectly, and that 95% of this agriculture is primarily rainfed [6]. This highlights the linkage between water use in agriculture and food and nutrition security and explains why agriculture remains the main vehicle for addressing food and nutrition security in poor rural households. Approaches to dealing with poverty and hunger in SSA have hitherto mainly focused on food production under water scarce conditions [6]. While this approach indirectly allowed for nutrition security to be addressed, it was not adequate, because agriculture and nutrition research occurred in silos. Given that nutrition security is the basis upon which human health and well-being are built [7], it is important to design a transdisciplinary approach with scientific and social credibility, especially as we move into the post-2015 era of Sustainable Development Goals (SDGs) [8]. Such multifunctional agricultural systems have been reported to have had more success in delivering on nutrition, health and income goals [9].

There is a need for a paradigm shift in order to effectively deliver on the twin challenges of food and nutrition security. Part of this means re-thinking the indices that have been used by researchers and development agencies in assessing food and nutrition goals and assessing their impacts on human health. Previous studies have focussed separately on either crop production (food availability) or food access or nutrition [8]. Thus, food production, nutrition and human health have been addressed separately using different indices, with the balance tilting in favour of food production. In order to meaningfully address food and nutrition security, there is a need for indices that combine aspects of production, access and nutrition. This could also encourage transdisciplinary research between agricultural scientists, nutritionists and dieticians. In doing so, recommendations can be generated that empower rural farmers so that they get the most (biomass and nutrition) per unit drop of water used—nutritional water productivity (NWP) [10]. According to Renault and Wallender [10], NWP, defined as nutritional value per unit of water, offers more meaning in linking crop water productivity, food production to malnutrition and poverty. The potential for application for NWP was highlighted by Wenhold *et al.* [11] in their report on water use and nutrient content. The study [11] made significant progress in establishing a baseline for future studies on nutritional water productivity of crops.

The review introduces the water-food-nutrition-health nexus as a way of planning agriculture based strategies for improving human nutrition and health in poor rural households. It hypothesizes that water, agriculture, nutrition and human health are intricately linked to each other—a nexus. In addition, it suggests that such linkages should be made explicit in planning agricultural interventions aimed at improving food and nutrition security and human health among the poor. Furthermore, recognition of these linkages entails transdisciplinary research. Such transdisciplinary research needs to be supported by the development of appropriate metrics that can articulate components of the linkage. In this regard, the review suggests the use of nutritional water productivity as a suitable index for assessing the contribution of water use and agriculture to food and nutrition security as a starting point.

The focus on neglected and underutilised crop species (NUCS), as an alternative to major crops, is meant to address the need for dietary diversity in poor rural communities. Within the scope of this review, NUCS are defined as those crops that have not been previously categorized as major crops, are currently under-researched or have not been the subject of major research projects, have low levels of utilisation and are mainly confined to niche agroecological areas [4].

## 2. Water

The role of water in achieving food and nutrition security for improved nutrition and human health cannot be understated. Water is essential to food and nutrition security through its linkages with all aspects related to economic access to food [12]. While the role of water in the provision of food and nutrition transcends many sectors, this review focusses primarily on the linkages between water and agriculture. Sufficient and quality water is critical for agricultural production [12] and achieving food and nutrition security.

Options for increasing agricultural production to meet the growing food demand include increasing (i) land area under production; (ii) yield, through crop improvement, plant biotechnology or other alternative methods; and (iii) output per unit input (productivity) on existing land area [13]. Agricultural productivity in SSA is currently low and remains far below yield potentials [14], suggesting that there is need to improve current levels of productivity on existing land area. Reports also suggest that poor rural farmers in SSA cannot afford inputs such as fertilizers, chemicals and herbicides to improve their productivity [15]. This may, in part, explain current low levels of productivity. In such instances, it may be more effective to develop agroecological approaches to crop husbandry and income generation opportunities so that farmers can afford inputs to achieving more sustainable systems (see [16,17,18]).

This review focuses on the third option with a particular emphasis on water as an integral input in agriculture. Global reports indicate that agriculture is the biggest water user, accounting for 60%–90% of fresh water withdrawals [19]. Water will be a major constraint for agriculture especially in SSA where rainfall is generally low and the population is increasing rapidly [20]. Recent reports on climate change [21] have confirmed this with indications that climate change impacts in SSA will mainly be felt through changes in rainfall and water availability. This has placed agriculture in a situation where any increases in agricultural production cannot be met with corresponding increases in water use.

This has brought about the slogan “more crop per drop” [22,23,24,25,26,27]. Increasing agricultural production without increasing water use will contribute to sustainability by ensuring food and nutrition security now and in the future without further threatening scarce and limited water resources.

## 3. Agriculture

In crop production, water use is often equated to evapotranspiration (ET) which is the combined loss of water from the soil surface by evaporation (E) and from the plant by transpiration (T) [28]. Evaporation and transpiration are the two processes where water is actually depleted from the soil; other outflows such as runoff and drainage are not considered as depletion as water is captured in other sinks such as ground water, saline water bodies and oceans [23].

Globally, rainfall contributes about 80% of water that is lost through evapotranspiration while 20% is drawn from water bodies for irrigation purposes [29]. Climate change and variability are expected to significantly alter this situation through changes in rainfall patterns with significant impacts on agriculture [12]. This threatens the role of agriculture in providing food and nutrition security, especially in water scarce areas. Ali and Talukder [30] suggested that improving productivity under rainfed conditions would decrease irrigation withdrawals and ultimately decrease pressure on fresh water resources. This would include adopting crops that thrive under rainfed conditions reducing the need for irrigation and adopting strategies that reduce the amount of water used by crops during production. It is important to note that these strategies are at the crop interphase and do not consider nutritional value which is key to nutrition security.

### 3.1. Crop Water Use

Crop water use refers to the amount of water lost by the crop through evaporation and transpiration (evapotranspiration) in exchange for biomass accumulation. Due to the combination of the processes of evaporation and transpiration, some authors refer to crop water use as crop ET [31,32]. Both processes are driven by point weather conditions, with some meteorological variables having a direct influence (wind, radiation, temperature and humidity) [28]. There are various methods of determining ET, which can be grouped into hydrological (e.g., soil water balance and lysimetric measurements) and micrometeorological techniques (e.g., eddy covariance, the Bowen ratio-energy balance and the temperature difference method). Hydrological approaches are often the most practical approach to determine ET under field conditions [28], and consider incoming and outgoing water fluxes [28]:

(1) ET = Irrigation + Rainfall − Runoff − Deep percolation + Capillary rise ± changes in subsurface flow ± changes in soil water content

Fluxes such as runoff, deep percolation, capillary rise and changes in subsurface flow are challenging to determine accurately under field conditions [28]. The published water use values of some crops suggest that vegetables such as carrots (*Daucus carota*) and garden beans (*Phaseolus vulgaris*) use the least amount of water (200 and 320 mm, respectively) while groundnut (*Arachis hypogaea*), soybean (*Glycine max*) and maize (*Zea mays*) are among the crops with high water use (700‒1000 mm) [11]. It is important to note that these values were obtained at a specific location and may not be applicable across various locations due to differences in weather conditions.

Water use values also differ with crop management and genotype. Efforts to determine water use of crops have been linked to relating it to biomass production and ultimately crop yield. Notably, these efforts have not focussed much on relating crop biomass and yield to the provision of nutrition for improved human health. They have assumed that more biomass and yield translate to more nutrition.

### 3.2. Improving Crop Water Productivity

There is a need to improve water productivity under rainfed production [30]. This will benefit the majority of SSA’s population (>70%) that relies on rainfed agriculture as the primary source of livelihood [5]. Despite the limitations in resources suffered by farmers across SSA, there is high confidence that productivity levels can be improved [33]. Currently, yields in the USA and Europe are approximately 200%–300% higher than yields achieved in SSA [34]. The huge yield gap is partly due to poor agronomic practices and limited use of improved crop varieties in SSA. The USA and Europe also have higher water productivity (~2 kg·m^−3^) compared to SSA (~0.2 kg·m^−3^). The disparity between these yield and water productivity values creates an opportunity for interventions to narrow the gap. The approaches taken so far have included adopting better agronomic practices, promoting use of improved varieties and improved water productivity. These strategies are not linked to increasing production of nutrient dense crops. Strategies to improve yield and water productivity should also consider increasing production of several nutrient dense crops in order to address nutrition.

There are possible crops that could be recommended in SSA where food and nutritional insecurity are currently high [11]. Among the vegetable crops, black nightshade (*Solanum nigrum*) and cleome (*Cleome gynandra*) have low water use and high water use efficiency (37–300 mm and 60 kg·ha^−1^·mm^−1^) [11]. Among the legumes, groundnuts (*Arachis hypogea*) yield the most (2–4.8 t·ha^−1^) but require more water (800 mm) compared to cowpea (*Vigna unguiculata)*, which yields 2.6–3.9 t·ha^−1^ and uses about 600 mm of water during the growing season. The same difference was observed for maize (*Zea maize*) and grain sorghum (*Sorghum bicolor*). Water use of grain sorghum was shown to be lower than that of maize, but they shared the same range of WUE (6–10 kg·ha^−1^·mm^−1^) due to sorghum’s lower grain yields [11]. Most of the crops reported to have lower water requirements in the different categories are underutilised crops of African origin or African traditional varieties. They thrive well under SSA conditions and with improved agronomic practices and improved varieties there is potential that these crops could contribute to future food security, especially under rainfed conditions [35]. While it is acknowledged that increasing yields and reducing crop water use is of great importance, there is a need to link crop water productivity with nutrition.

### 3.3. Relating Water Use to Dry Matter Production in Crops

While the focus has been on increasing dry matter production in crops, it is the amount of water required that agricultural scientists are concerned about. This is because water is a finite resource and its efficient use in crop production is of primary concern [36]. It also addresses the “more crop per drop” slogan that is being promoted by agricultural and water practitioners. The history of efficient use of water dates back to the 1950s [37]. Then, since irrigation was considered responsible for the highest water withdrawals, irrigation scientists attempted to control and manage irrigation water by introducing an irrigation efficiency (IE) ratio defined as water consumed by crops of an irrigated farm to the water diverted from the source [33]:

(2) $$IE=\frac{\text{water consumed}}{\text{water diverted}}$$

where, IE is irrigation efficiency, water consumed is the volume of water consumed by crops on an irrigated farm and water diverted is the volume of water diverted from the source for that purpose [36,38,39]. The IE ratio was from an engineering perspective and facilitated better water allocation from water sources to farms. It did not account for crop yield and water used to produce the crop, failing to describe the relationship between yield output per unit of water.

The relationship between yield output per unit of water started being investigated before the 19th century as a measure of drought tolerance [36]. It was termed efficiency of transpiration, defined as dry matter produced per unit water transpired:

(3) $$TE=\frac{B}{T}$$

where, TE is efficiency of transpiration (g·kg^−1^ water), B is the total dry matter produced (g) and T is mass of water transpired (kg) [40]. From a strictly engineering perspective, this was more of an efficiency index as it produced a unitless ratio. In the late 1950s, the term employed to describe the relationship between yield output per unit of water was water use efficiency (WUE) [41]. It was defined as mass of dry matter produced per unit volume of water evapotranspired:

(4) $$\mathrm{WUE}=\frac{B}{\mathrm{ET}}$$

where, WUE (g·mm^−1^) is water use efficiency, B is total dry matter produced (g) and ET is amount of water evapotranspired (mm). Viets [41] justified the term WUE as being more appropriate since it emphasises water whose efficient use was the subject of interest. Water use efficiency was then widely used under field conditions to describe the ratio between yield/biomass per unit water used. However, depending on scope and scale of studies, WUE was understood, interpreted and calculated differently [32,42,43,44,45]. Molden [46] introduced the term water productivity (WP) to describe the ratio between yield output per unit of water. Productivity of water emerged during a System-Wide Initiative on Water Management (SWIM), to develop standardised water accounting procedures [46]. Since then the terms WUE and WP have been used interchangeably, creating much confusion for researchers, practitioners and policy makers.

### 3.4. Water Use Efficiency and Water Productivity

While the terms WUE and WP seek to address the notion of “more crop per drop”, they are now being used interchangeably and seemingly lack common definition. The efforts in distinguishing WP and WUE sparked created debate with several papers being written on the subject, where irrigation engineers, crop physiologists and water managers hold different perspectives. Molden *et al.* [23] proposed a common conceptual framework for communicating water productivity. They highlighted the importance of water accounting at various scales of interest. WUE calculated as:

(5) $$\frac{B/Y}{\text{water applied}}$$

where B/Y is biomass (B) or yield (Y) (g) and water applied is water into the system as precipitation and/or irrigation (mm) or:

(6) $$\frac{B/Y}{\mathrm{ET}}$$

where B/Y is biomass or yield (g) and ET in mm (calculated as the residual of precipitation + rainfall ± changes in soil water content) are efficiency ratios to describe the ability of crops to utilize water made available to them [47].

The denominator in Equations (5) and (6) (water applied and ET) assume that water entering the field through irrigation and precipitation is all taken up via crop evapotranspiration. These do not account for other water movements in and out of the system such as runoff, deep percolation, capillary rise and changes in subsurface flow. This may be attributed to the fact raised by Allen *et al.* [28] that water fluxes were challenging to quantify; hence, in many cases, authors assume the effect of other water fluxes to be negligible. While this approach may be justified, it posed difficulties in its application as a comparative measure of efficacy [47]. This was reflected in the scoping study where WUE values derived from various studies showed a wide range. A classic example was for carrots where an experiment conducted in South Africa reported WUE values of 131–148 kg·ha^−1^·mm^−1^ and experiments conducted in Chile reported WUE of 19.4–28.3 kg·ha^−1^·mm^−1^ for the same crop [11].

On the other hand, WP calculated as

(7) $$\mathrm{WP}=\frac{\mathrm{Ya}}{\mathrm{ETa}}$$

where WP is water productivity (kg·m^−3^), Y_a_ is the actual yield (kg) and ET_a_ is the actual evapotranspiration (mm·ha^−1^ or m^3^) or water consumed [48,49] is a true efficacy parameter of the crop production process. In this case, the denominator only considers water consumed by the crop for biomass production [47]. When calculating WP only, the water consumed by the crop through evaporation and transpiration is considered. Complex water flows in and out of the system are accounted for in order to determine the actual volume of water consumed by the crop. One advantage of WP is that unlike WUE, its values are more conservative, hence, comparable across temporal and spatial scales [48,50]. However, these arguments, while valid, tend to narrow the scope of WP.

Molden [29] simplified WP by defining it as the benefits derived from a unit of water, thus, from a holistic point of view, WP can be used to analyse water management. More recently, the High Level Panel of Experts (HLPE) on Food and Nutrition also weighed in on the subject in their report on linking water to food and nutrition [12]. While concurring with Molden [29], they reasoned that owing to its origins in agronomic and economic sciences, water productivity was more an output-centred concept. In this way, the output could vary from crop yield, monetary value, or nutritional value per unit of water [12], making WP a more useful parameter for water management. For the purposes of this review, the reasoning of the HLPE is followed, because it provides a basis for linking water use in agriculture to nutrition.

### 3.5. Linking Water Productivity to Nutrition—Nutritional Water Productivity

The progress made over the last few decades by agricultural scientists towards water saving strategies is laudable [11,12,13,14,15,16,17,18,19,20,21,22,23]. Irrigation scientists have designed more efficient irrigation systems, breeders have identified and bred high water use efficient crops and crop scientists have identified more water productive cropping and field management systems. While these efforts towards improving water productivity remain commendable, the question remains—“is this enough?” Water scarcity is not the only global challenge that needs to be addressed. Between 30% and 40% of the world population is malnourished; they experience some form of undernutrition, are overweight or obese, or have some sort of micronutrient deficiency [7]. As such, malnutrition is a problem that is currently affecting nearly every country in the world although it is more prevalent in SSA.

Addressing malnutrition should focus on nutritional value and quality of agricultural produce and diets as most nutrients come from crops [51]. Looking at the United Nations’ [52] proposed SDG 2 (End hunger, achieve food security and improved nutrition and ensure sustainable food production by 2030), it is clear that while the “more crop per drop” approach addresses ending hunger and achieving food security, it is silent on issues of nutrition. It is directly linked with increasing food availability and access while silent on utilisation, hence, ultimately failing to achieve sustainable food production. Sustainable food production describes the capability of agriculture over time to provide sufficient and nutritious food at all times in ways that are economically efficient, socially responsible, and environmentally sound [53]. The water productivity approach focusses on dry matter production but does not account for nutritional content of the biomass produced. As such, efforts towards increasing food and nutrition security will possibly improve physical and economic access to sufficient food, from the production perspective. This will be in addition to efficient utilisation of water resources by agriculture. However, the WP approach falls short of addressing aspects related to provision of “nutritious food capable of meeting their dietary requirements and food preferences for an active and healthy life”. Therefore, there is a need to link the concept of “more crop per drop” with aspects of nutritional value. This could provide a holistic approach to linking water, agriculture and nutrition with improving human health.

In this regard, achieving sustainable food production should also focus on dietary requirements and their relation to scarce water resources [10]. This is especially true for the rural poor where the link between water and food is a crucial link for nutrition and livelihood security [24]. Within these communities, improved nutrition will lead to improved health, development and productivity [54]. This was proposed by the Stockholm International Water Institute (SIWI) and the International Water Management Institute [24] under the notion of “more nutrition per drop”. Renault and Wallender [10] had earlier identified this relationship and termed it nutritional water productivity (NWP). By definition, NWP is nutritional content per volume of water consumed [10]. Hence,

(8) NWP = (Ya/ETa) × NP

where NWP is the nutritional water productivity (nutrition m^−3^ of water evapotranspired), Y_a_ the actual harvested yield (kg·ha^−1^), ETa the actual evapotranspiration (m^3^·ha^−1^), and NP is the nutritional content per kg of product (nutrition unit·kg^−1^). A closer look at Equation (8) shows Y_a_/ET_a_ = WP as described in Equation (7).

When determining nutritional value there is need to consider many other nutritional components of food [10]. There is a dearth of empirical information describing NWP for a range of crops. Where available, values of NWP are calculated using WP and NP values obtained from separate studies. For example, in the scoping study by Wenhold *et al.* [11], published international benchmarks for energy, protein and fat values of selected crops from Canada, Ghana and the USA together with WUE values obtained separately were used to calculate NWP (Table 1). Another concern is the values Wenhold *et al.* [11] refer to as WP. Were they actually WP values or were they a combination of WP and WUE values but for the sake of consistency were all referred to as WP? As mentioned earlier WUE values are less conservative and when used to determine NWP can increase inaccuracy. In addition, nutritional composition values were obtained from crops grown under different conditions and at different water contents. This casts doubt on the conservativeness of NWP values. However, the importance of the study [11] was not in calculating NWP, but rather in highlighting the linkages between water, agriculture and nutrition (Table 1).

Based on South African benchmarked values of macronutrient water productivities, among the vegetable crops, sweet potatoes (*Ipomoea batatas*), carrots, pumpkin leaves (*Cucurbita pepo*) and amaranth (*Amaranthus* sp.) showed high efficiency in terms of water consumed per energy and carbohydrates produced (>20 MJ·m^−3^ and > 300 g·m^−3^). Amaranth and pumpkin leaves were efficient in terms of water consumed per protein produced (>500 g·m^−3^). Legumes generally did not show a wide range in energy and protein water productivities, but soybeans, lentils (*Lens culinaris*), groundnuts and dry beans (*Phaseolus vulgaris*) were the most efficient (>100 g·m^−3^) with respect to protein water productivity. Among cereal crops, only maize NWP values were estimated. Maize was not as efficient as sweet potatoes, carrots, pumpkin leaves and amaranth with respect to energy and protein water productivity (17 MJ·m^−3^ and 132 g·m^−3^, respectively). Maize was, however, an efficient synthesiser of carbohydrates (672 g·m^−3^). Wenhold *et al.* [11] also calculated NWP for minerals and vitamins for selected crops. It was apparent that vegetable crops were the most efficient. Their results highlighted the potential of amaranth and pumpkin leaves with respect to their high NWP. Coincidentally, these crops are currently underutilised.

**Table 1:** Nutrient content and nutritional water productivity (NWP) (macronutrients) (Adapted from [11]).

Food Group	Product	Nutrient			NWP			Source *
		^x^E	^y^P	^z^F	^x^E	^y^P	^z^F	
		kcal·kg^−1^	g·kg^−1^		kcal·m^−3^	g·m^−3^		
Vegetables	Tomatoes	184	8	1	1416	65	11	[10]
	Carrots	25	10		2174	87		[55]
	Cabbage	250	14		3289	89		[55]
	Pepper	200	12		38	2		[55]
	Onions	331	12		880	31		[56]
Legumes	Groundnut	6067	283	426	2382	111	206	[10]
	Peas	2720	229		8889	748		[55]
	Green beans	330	24		935	68		[55]
	Soybean	3470			2828	304		[55]
		4160		200	956.8	83.95	46	[56]
Grain	Maize	2738	55	12	3856	77	17	[10]
		3270	85		8583	223		[55]
		2738	55	12	547.7	11	2	[56]

^x^E = Energy; ^y^P = Protein; ^z^F = Fats; * [10] (USA); [55] (Canada); [56] (Ghana).

Unlike WP, which only focuses on increasing food availability and access, NWP addresses availability, access and utilisation components of food security. It also provides a linkage for water, agriculture and nutrition, making NWP a more useful index for evaluating impacts of agriculture on food and nutrition security. There is a need to promote the use of NWP in studies using agriculture to address nutrition and health aspects. Concurrently, there is also a need for transdisciplinary dialogue and studies to develop more such appropriate metrics that can be useful to operationalizing transdisciplinary efforts linking water, agriculture and nutrition.

## 4. Food for Nutrition

While there has been much effort towards combating food and nutritional insecurity, the balance has been tilted more in favour of food production than nutrition. The concept of “food and nutrition security” in itself may have unintentionally led to the two being treated as separate, with more focus on food production. The fact that agriculture underpins rural livelihoods and is inextricably linked to rural development explains why, over the past decades, large investments have been made in agriculture. In addition, such agricultural production is assessed mostly by metrics of crop yield, economic output [57] and resource use efficiency/productivity, which do not address the nutritional value associated with the crop yield [58]. The evolution of the concept of NWP and the limited literature on its application speaks volumes about the limitations of current agricultural interventions to deliver nutrition outcomes. The IAASTD [57] highlighted the need for a holistic and multi-functional approach as well as a need for an agro-ecological approach to farming. This, they argued, could assist in transforming agriculture to deliver on issues of food, nutrition and sustainability.

Although there are now growing calls for agriculture to deliver nutritional goals [59], there has been a lag in integrating issues of nutrition into planning and evaluation of agricultural and food systems and policies [58]. In order to achieve such integration, there is a need for a paradigm shift with regards to food and nutrition security. Part of this requires revising the notion of “food and nutrition” and focussing rather on “food for nutrition”. The latter recognises the crucial link that food, in the context of this review derived from crop production, is the primary source of nutrition in human diets. A draft definition of nutrition security states that “nutrition security exists when all people at all times consume food of sufficient quantity and quality in terms of variety, diversity, nutrient content and safety to meet their dietary needs and food preferences for an active and healthy life style, coupled with a sanitary environment, adequate health, education and care” [60]. This involves obtaining, utilising and absorbing nutrients that are required for normal growth, health and social wellbeing [54]. Although there is now emerging interest in addressing nutrition, malnutrition still remains a significant development challenge and multidimensional problem in infants, young children, and women [54].

Malnutrition continues to cause poor health, morbidity and mortality from childhood through to adulthood, affecting social and economic development among the rural poor. About 38% of children in least developed countries suffer stunting due to undernutrition; this could negatively impact on cognitive development during early childhood development [61]. Burchi *et al.* [62] emphasised the vital role of nutrition for growth, cognitive development and health, suggesting that there could be significant consequences for children who experienced undernutrition in the first 1000 days of their lives. They were likely to perform poorly in math and science subjects in the future [61]; this in itself would help to perpetuate poverty in poor rural communities. In this regard, it can be hypothesised that breaking the cycle of malnutrition during early childhood development could go some way towards breaking the cycle of intergenerational poverty among the rural poor. This is especially true for SSA where social inequality is still rife and continues to perpetuate poverty among the rural poor [63]. Poor rural farmers lack the privilege of having access to diverse food sources and choices [54]. Where food sources are available and accessible, it is their utilisation that limits poor rural communities from deriving the nutritional benefits therein.

Nutrition is inextricably linked to utilisation. Nordhagen [64] suggested that in order to achieve food and nutrition security, policy makers must look at agriculture as not merely the provider of food, but rather as the provider of nutrition in human diets—food for nutrition. This is because nutrition is seldom about adequate food supply/availability but rather impeded utilisation which is often associated with poverty and lack of education [64]. As such, efforts aimed at addressing nutritional insecurity should not be merely technological interventions aimed at boosting productivity, but should also consider social anthropogenic issues that influence food utilisation such as sources of food and reasons for food choices consumed by poor rural households.

### 4.1. Food Intake, Sources of Food in Poor Rural Households

As previously highlighted, much of the effort directed towards achieving food and nutrition security has been crop-centric and biased towards increasing food availability and accessibility with less attention paid to nutrition. Food availability and access are some of the factors linked to food and nutrition security of rural households [11]. However, knowledge of food sources consumed by poor rural households is essential to developing appropriate food-based interventions for addressing malnutrition (under- and over-nutrition) in rural households. While it may be difficult to clearly define a food basket of food consumed by rural households, it is clear that diets among this group are mainly starch (cereals and root and tuber crops) based [11]. Other food groups such as legumes, fruit and vegetables do not feature prominently in diets of poor rural households. This apparent lack of dietary diversity could explain some of the underlying reasons for undernutrition amongst the rural poor.

Agriculture, because of its potential to produce nutrient dense foods, can play an important role in addressing dietary diversity [54]. Diverse cropping systems and inclusion of underutilised crops in food for nutrition interventions could address dietary diversity and contribute to nutritional goals. Multicrop systems and underutilised crops have historically played a crucial role in the diets of rural people [65,66]. Bezner *et al.* [67] reported that intercropping of maize and legumes in Malawi for improved dietary diversity resulted in improved levels of nutrition in children under five years. In this instance, agricultural interventions were also coupled with nutrition education at the community level [67]. Underutilised crops which are mostly affordable and often free, if sourced from the wild, are often nutrient dense. However, a lack of education on their nutritional value and stigma remain an obstacle to improving their utilisation amongst poor rural households. Therefore, in addition to addressing scientific knowledge gaps on food for nutrition, there are social barriers that need to be addressed to ensure uptake and utilisation by poor rural communities. This emphasises the need to understand reasons for food choices in poor rural households.

### 4.2. Reasons for Food Choices in Poor Rural Households

Reasons for food choices reflect the various causes that, either separately or synergistically, influence why people consume certain foods and not others. Reasons for food choices are more than a simple matter of nutritional value [68]. Each time people choose food, they bring their past food choices, events, experiences, thoughts and feelings as well as historical context to the fore [69]. This salient observation is important in highlighting why people eat what they eat as a complexity. In addition, reasons for food choices exist at various levels and are influenced by a range of factors such as demographics, health awareness [68], availability, affordability, taste and cultural preferences among others. Whilst availability and affordability can be dealt with from a technical viewpoint, it is the other variables and often dynamic reasons that present challenges.

While noting the shortfalls of previous research in interrelating the variables influencing food choices, Sun [68] suggested that research needed to be dynamic and to transcend the boundaries of nutritional value. This would allow researchers to grasp the dynamic nature of reasons behind food choices. Wenhold *et al.* [11] emphasised that understanding reasons for food choices was important as they were directly linked to the food and nutrition security of rural households. This also included gaining knowledge of why rural households used certain foods more frequently than others [11]; this would be essential for coordinating an effective food for nutrition programme. The authors [10] suggested that while macro-, meso- and micro-level factors influenced reasons for food choices, it was the latter that required more in-depth research and case studies in order to make meaningful breakthroughs.

The different issues regarding food for nutrition, food sources and reasons for food choices present a case for a multidisciplinary approach to address nutrition security in poor rural households. Agriculture has a role to play through providing food for nutrition. It also has a role to play through increasing dietary diversity and giving people more choices. In addition, agriculture can improve household incomes and allow people to afford other nutritious foods. Nutrition education is still needed to allow people to exercise their choices and to utilise the food from agriculture in order to derive the nutrition required for a healthy lifestyle.

### 4.3. Underutilised Crops: Potential to Contribute to Food and Nutrition Security

As we continue to re-think strategy, focus should also be on diversifying the food basket in order to create dietary diversity in poor rural households. Diets of poor rural households are essentially starch-based and lack in protein, vitamins and other mineral nutrients. The reasons for this include limited food sources and choices [11,70] mainly associated with poverty. Fanzo [54] stated that dietary diversity was an integral component of a quality diet; both diversity and quality are key to achieving food and nutritional goals. In addition, consumption of a diversified diet ensures adequate provision of essential nutrients and health enhancers [71,72,73,74,75]. However, access to a diversified diet is often hampered by a lack of understanding on its effects on human health and affordability. While the latter is partly true, this thinking fails to recognise the rich tapestry of agro-biodiversity that exists within rural landscapes, which could be a source of sustainable dietary diversity for poor rural households at little to no cost. In addition, a lack of skills and understanding also hampers the effective utilization of such agro-biodiversity [76,77]. This concurs with suggestions by Schultz [78] and Backeberg [79] that there was a need for human capacity development to support and sustain agricultural interventions. Such capacity development could be in the form of participatory approaches as exemplified by the work reported by Tchoundjeu *et al.* [77] on domestication of indigenous fruit trees. The findings of Wenhold *et al.* [11] (Table 1) also justify the need to conduct more research on underutilised crops for purposes of contributing to food and nutrition security.

Neglected and underutilised crops species (NUCS) have potential to improve access to and availability of nutritious food for poor rural households. There currently exists no common definition of NUCS with variations in classification due to geography (underutilized/neglected where?), social (underutilized by whom?) and economic (to what extent) concerns [80]. While acknowledging these shortcomings, Chivenge *et al.* [4] defined NUCS as “crops that have not been previously classified as major crops, have previously been under-researched, currently occupy low levels of utilisation and are mainly confined to smallholder farming areas”. Such crops may have potential to contribute to food and nutrition security of poor rural households and may be suited to the marginal production environments that typify most rural areas in SSA [4]. However, NUCS currently occupy low levels of utilization. Limitations in utilisation have been linked to limited research addressing basic aspects of their production, water use, nutritional value and seed systems [65]. There is a gap in agronomic information on the range of crops that could be utilised as food security crops [11]. This highlights the need for more research targeted at NUCS with a view to tapping into their potential to contribute to food and nutrition security in rural areas.

Indigenous fruit trees also form an important aspect of agro-biodiversity that is currently underutilised within SSA. A recent review by Chivenge *et al.* [4] noted that indigenous fruit trees which were often consumed by poor rural communities were highly nutritious. Indigenous fruit trees were reported to be rich sources of sugars, essential vitamins, minerals and essential oils [81,82]. However, their potential to contribute to food for nutrition and health was currently not being fully explored. Unlocking the value of NUCS requires multidisciplinary initiatives which can recognize the diverse roles that NUCS often play within the communities that have preserved them. Reports of work done in Cameroon to promote utilization of indigenous fruit trees provides some evidence of the transformation that can be achieved through multifunctional approaches [9,83].

Inclusion of NUCS will broaden the food basket hence influencing food choices, especially in rural communities where access to nutritious food is a major limitation. Within these communities, inclusion of NUCS would not only increase dietary diversity but would also increase dietary quality, hence contributing to improved human nutrition and health.

### 4.4. Competing Needs in Poor Rural Household

When considering food for nutrition, reasons for food choices and crop diversification in poor rural households, competing economic interests within poor rural households also require consideration. For example, farmers may grow a nutrient dense crop and opt to sell the crop as opposed to consuming it within the household. Other related competing interests in poor rural households include the need for energy production on the farm (manure for cooking, fuel wood needs, or commercial biofuel crops), which have an impact on competing water uses and on soil quality, all of which are interlinked This adds to the general complexity of understanding reasons for food choices in poor rural households and partly explains why technical interventions, alone, may not be successful. The occurrence of such competing needs also emphasises the need for multidisciplinary approaches that not only address biophysical constraints, but also socio-economic constraints.

## 5. Human Health and Well-Being

According to the World Health Organisation [84], “health is a state of complete physical, mental and social well-being and not merely the absence of disease or infirmity”; this definition has not been amended since 1948. The Global Food Report [7] emphasised good nutrition as being the basis of human health. This underscored the importance of nutrition to human health and well-being. In addition to nutrition, several other factors are known to influence human health and these include background, culture/lifestyle, and economic and social conditions. Interestingly, these factors can be linked to factors that influence reasons for food choices and nutrition thus reaffirming the critical role of nutrition to human health. It is for this reason that we are interested in nutrition [70], hence, human health and well-being are the primary end goal for food for nutrition.

Nutrition has always been a key development indicator. The consequences of inadequate nutrition include poor human health, well-being, productivity, livelihood and consequently a stagnation in national development [85]. For developing regions such as SSA, this can have huge negative impacts on economic development and poverty alleviation efforts. This justifies the need for governments within SSA to invest in food for nutrition as a strategy for improving human health and well-being among the rural poor. The economic justifications for investments in nutrition have long been established. Behrman [86] reviewed the economic justification for developing countries to invest in nutrition and found that improved nutrition resulted in improved health, labour productivity and general upliftment of the poor. Investments in food for nutrition which targeted the rural poor were a cost effective use of national resources and led to reduced social and financial losses associated with poor health resulting from malnutrition [86].

The consequences of malnutrition are even more severe on the health and well-being of vulnerable groups which include children under five years and breastfeeding women. For children under five years, inadequate nutrition is a major contributing factor to child mortality; undernutrition accounts for more than 33% of child mortality in low-income countries [87]. Adequate nutrition is essential for cognitive development during early childhood development, and hence educational success, both of which are important determinants of labour productivity and economic growth [61,87]. Globally, an estimated 162 million children remain moderately or severely stunted, an indication of chronic undernutrition [88,89], with most of these children in the developing world.

In pregnant women, malnutrition has been associated with low birth weight and varying levels of mental retardation in offspring. Intelligent quotients (IQs) of infants with low birth weight have been reported to be about five points lower than for normal weight infants [90]; thus, setting in motion an unfortunate cycle which can perpetuate poverty [91]. The effects of malnutrition on pregnant and breastfeeding women as well as on infants and children further justify the need for robust food for nutrition programmes targeted at poor rural communities. Lack of adequate nutrition and the consequences thereof on human health can have the effect of perpetuating poverty, making it difficult for generations of poor rural people to ever escape poverty. In order to achieve food for nutrition programmes that can effectively improve human health and well-being among the rural poor, there is a need to fully recognise the various linkages that contribute to this end goal.

In this regard, realising the crucial linkages between water, agriculture, nutrition and human health is essential to achieving the endpoint of a healthy nation. Hawkes and Ruel [92] described these linkages as mutual in that while water and agriculture affected nutrition and health, the latter two also affected agriculture. However, the linkages between water, agriculture, nutrition and health are still not well-understood. This may be because traditionally these sectors have used, and still continue to use, distinct indices to assess their impacts on human well-being. The use of an index such as NWP offers the possibility of linking water to agriculture and nutrition via the food for nutrition paradigm.

## 6. Water-Food-Nutrition-Health Nexus

In developing countries, the goal of the water, agriculture, nutrition and health sectors is to improve the standard of living of people, especially historically disadvantaged poor rural communities. Despite this shared goal, agriculture has seldom been openly set out to address nutrition and health challenges [93]. This is because the linkages between agriculture and nutrition have not always been explicit in planning. There is a need for a paradigm shift in terms of how we continue to deal with food and nutrition security. In the past decades, the “green revolution” made huge strides in boosting food production in much of SSA [90]; however, an unforeseen problem emerged in the form of inadequate nutrition. While investments in agriculture increased crop production, mainly for cereal crops, this was accompanied by declining crop diversification, production and utilisation of traditional food crops (e.g., legumes and leafy vegetables) with high nutrient density [90]. Although unintended, this reduced dietary diversity and nutrition, especially for poor rural households. The goals that drove the “green revolution” did not explicitly include adequate nutrition as an output from agriculture. If the endpoint to agriculture is improved human health anchored on good nutrition, it follows that the role of agriculture in providing adequate nutrition must be explicit in planning. Shifting agriculture to focus on “food for nutrition” would establish the crucial linkages between food and its role as the primary source of nutrition in human diets. In addition, given the water-food nexus, such agriculture should also be water-smart [94].

The lack of explicit linkages between water, agriculture, nutrition and health is not isolated to agricultural planning alone. A paradigm shift is also required with regards to how we deal with nutritional challenges. In the past, the response from human nutritionists was rather medical in that it viewed malnutrition as a “disease” that needed to be “treated” [90]. Consequently, intervention programs spearheaded the use of supplements or food fortification programs to “treat” malnutrition [90]. In cases where crops were short-listed to combat malnutrition, only a few were singled out for their ability to deal with specific nutrient deficiencies (e.g., orange fleshed sweet potato to tackle Vitamin A deficiency). Although these nutritional interventions have registered successes in the short-term, they have failed in the long-term due to various social, policy and technical problems which affected their sustainability [95,96,97]. Most importantly, these approaches did not fully embrace the opportunities that exist along the agricultural value chain to improve human nutrition and health. Including nutrition on the agricultural value chain would ensure that nutritious foods are more available and affordable for poor rural households; this would also improve utilisation wherein nutrition lies. The need to consider the interlinked value of agriculture and nutrition has been recognised for many years. For example, a 1992 global conference on nutrition concluded that “most nutrition programs directed at eliminating malnutrition do not consider using agriculture as a primary weapon in their arsenal against this public health crisis” [98]. In many agrarian countries and rural communities in particular, agriculture remains best placed to effectively deliver nutritional outcomes and improve the health status of the rural poor. In order to achieve this, the linkages between water, agriculture, nutrition and health must be made explicit in planning agriculture-based interventions for improving nutrition and health of the rural poor.

While the linkages between water, agriculture, nutrition and health have always existed, it is only recently that they have become more recognised. The agriculture-nutrition-health nexus which highlights these linkages only came to prominence in 2011 [93]. It called for coordinated action in agriculture, nutrition and health planning and implementation. Proponents suggested that, if fully adopted and implemented, it could have significant positive impacts for food and nutrition security and development as well as improving the health status of the rural poor and women [54]. Some progress has been made in this regard. Since 2011, several multilateral agencies have started to realign their programs in line with the agriculture-nutrition-health nexus [93]. In Africa, the New Partnership for Africa’s Development (NEPAD) is currently exploring opportunities to fully integrate the agriculture-nutrition-health nexus into Africa’s agriculture roadmap—the Comprehensive Africa Agriculture Development Program (CAADP) framework [93]. While this process is on-going, some individual member states have also started to apply the agriculture-nutrition-health nexus to national planning. Uganda launched a Nutrition Action Plan (2011–2016), while Malawi has brought together policymakers and planners in the agriculture, nutrition, and health sectors to coordinate and integrate their activities on the basis of the agriculture-nutrition-health nexus [93]. Although the agriculture-nutrition-health nexus is now being recognised, there is currently scant empirical evidence on how these linkages work [93,99,100].

There are, however, limitations to the agriculture-nutrition-health nexus in that the role of water is not made explicit. Some might contend that water already features in all three, hence, there is no need to explicitly mention it. However, it is also for that very reason that water should be included in the nexus. This review has so far established the water-food nexus highlighting that food production is inextricably linked to water. In addition to the linkages between water and food production, water also plays significant roles in nutrition and hygiene from a water and sanitation perspective. The HLPE [12] affirmed the crucial linkages between water and food and nutrition security and highlighted the challenges of securing food and nutrition security under increasingly water scarce conditions. Recognising this important linkage is more essential to securing food and nutrition security in SSA where water scarcity (physical and economic) is the major limitation to crop production. This is especially true under rainfed agriculture which is practiced by more than 70% of SSA’s population. The need to include water alongside agriculture, nutrition and health has previously been suggested by several authors [12,54,92,101]. Furthermore, across SSA the impacts of climate change and variability on food production will mainly be felt through water. As such, including water in the nexus would ensure that agriculture becomes resilient through water-smart agricultural practices. This review therefore proposes that the agriculture-nutrition-health nexus be renamed the water-food-nutrition-health nexus. Compared to the former, the latter includes the water sector and focuses more on food for nutrition, thus making the roles of water and food for nutrition explicit.

In order to operationalise the water-food-nutrition-health nexus, there is a need for research on the nutritional and health impacts of water use for food production. To achieve this, more nutrition-relevant data need to be generated and collected from agricultural experiments, while nutritional and health indicators should be included in evaluations of agricultural programs [93]. There is also need to develop common indices that can be used to measure the health outcomes of agricultural interventions. In this regard, the use of NWP, if linked to health indicators, could prove instrumental in operationalising the water-food-nutrition-health nexus.

The objective of improving the well-being of poor communities requires a transdisciplinary approach that addresses all aspects of improving the quality of life [78,79]. Focus on agriculture alone tends to overate the value of land [78] at the expense of water, nutrition and health. They all require equal attention in order to transform the quality of life among poor communities. In this regard, adopting the water-food-nutrition-health nexus for planning rural development and food and nutrition security programmes could prove beneficial to SSA.

## 7. Conclusions

While progress has been made towards improving food security through improvements in crop production, the same cannot be said of nutrition security. Little attention has been paid to nutritional goals and linking them to agriculture programmes as well as the endpoint of improved human health and well-being in poor rural households. Consequently, poor rural households still suffer unacceptable levels of malnutrition despite the various interventions that have been made to improve their status. Inadequate nutrition in poor rural communities is partly associated with lack of dietary diversity and limited access to and non-availability of nutrient dense foods. Improving the utilisation of several NUCS could increase dietary diversity and increase access to nutrient dense foods in poor rural communities. The role of indigenous fruit trees, which also qualify as NUCS, in agroecology and income generation initiatives should be considered. Malnutrition, if left unchecked, could perpetuate poverty in poor rural communities. Programmes aimed at addressing food and nutrition security for improved human health have had limited successes. This is because of failure to clearly recognise the crucial linkages between water, agriculture, nutrition and health outcomes. There is a need for a paradigm shift which includes adopting the water-food-nutrition-health nexus approach. This would ensure that nutrition is made an explicit output in agricultural interventions. Developing appropriate metrics, such as NWP, which can be used to evaluate water, crop productivity, nutrition and health impacts would also go some way in achieving this. In order to operationalise the water-food-nutrition-health nexus, there is a need for interdisciplinary studies that can address the knowledge gap which is critical for formulating policy.
